# The role of AI-powered molecular profiling in the diagnosis and management of cancers of unknown primary: a case report and literature review

**DOI:** 10.3389/fonc.2026.1664264

**Published:** 2026-03-25

**Authors:** Abdullah Esmail, Hala Hassanain, Joanne Xiu, Maen Abdelrahim

**Affiliations:** 1 Section of Gastrointestinal Oncology, Houston Methodist Neal Cancer Center, Houston Methodist Hospital, Houston, TX, United States; 2 Charles W. Duncan Jr. Department of Medicine, Caris Life Sciences, Phoenix, AZ, United States; 3 Faculty of Medicine, The University of Jordan, Amman, Jordan

**Keywords:** case report, molecular intelligence, molecular profiling, prostate cancer, renal cell carcinoma

## Abstract

**Background:**

Molecular profiling (MP) and next-generation sequencing (NGS) have expanded the diagnostics evaluation of cancer by enabling genomic characterization that informs diagnosis and treatment selection. As the second leading cause of global mortality, cancer claimed nearly 10 million lives in 2020. Artificial intelligence (AI)-powered MP is increasingly recognized for its precision in diagnosing complex cancers, particularly cancers of unknown primary (CUP).

**Case presentation:**

We report a case of a 69-year-old male with a history of treated prostate cancer, initially diagnosed with CUP and suspected pancreaticobiliary malignancy. Conventional diagnostics, including imaging and immunohistochemistry, failed to identify the primary tumor, leading to ineffective therapies. AI-powered MP (Molecular Intelligence) revealed an 82% likelihood of renal cell carcinoma (RCC), prompting a targeted biopsy that confirmed RCC with sarcomatoid differentiation, enabling effective treatment with pembrolizumab and axitinib.

**Conclusions:**

This case highlights the potential role of AI-powered MP in resolving diagnostic uncertainty in CUP, where traditional methods like imaging and tumor markers are inconclusive. By identifying actionable biomarkers, MP facilitates personalized therapy, improving clinical outcomes. Further research is needed to validate the broader clinical utility of AI-driven MP as a decision-support tool for precision oncology.

## Introduction

Cancer remains the second leading cause of death worldwide and in the United States (US), with varying mortality rates mainly due to genetic and environmental factors. Recent studies reported more than 19.3 million new cancer cases per year (1). The incidence of cancer is constantly increasing worldwide, imposing a heavy burden on healthcare and necessitating new approaches to aid with early detection and treatment of cancer.

Cancers of unknown primary (CUP) are a diverse group of metastatic cancers without an identifiable primary site after adequate diagnostic evaluations (2). Historically, cancers of CUP accounted for 3–5% of all cancers, but recent data indicated a declining incidence rate, reaching 1–2%. The reasons for this decline remained speculative, potentially attributed to improved diagnostic techniques, such as molecular profiling, though multiple confounding factors, including variations in cancer registry reporting and diagnostic practices, complicated analysis (3). These trends underscored the ongoing challenge of accurately identifying CUP and the need for advanced tools like AI-powered molecular profiling to resolve diagnostic uncertainties (3). Traditional diagnostic methods, which rely heavily on imaging and histopathological examination often face limitations in accurately determining the primary origin of CUP (2, 4). In recent years, the field of oncology has witnessed significant advancements in molecular diagnostics, offering new diagnostic methods and insights into management (2, 4).

Cancer develops through a combination of somatic mutations, chromosomal aberrations, and epigenetic alterations, while germline mutations account for approximately 5-10%of all tumors.

assay is a commercially available multi-omics tumor profiling platform that integrated genomic, transcriptomic, and proteomic data. Molecular profiling (MP) involves analyzing tumors’ genetic and molecular characteristics to identify specific biomarkers, gene mutations, and other alterations. Technologists such as next-generation sequencing (NGS) and comprehensive genomic profiling (CGP) provide detailed genomic, transcriptomic, and proteomic data, which can be particularly useful in CUP cases for identifying tumor origin, guiding treatment decisions, and predicting prognosis and treatment response (5–7).

Artificial intelligence (AI) in healthcare plays a crucial role in the management of various fatal diseases, specifically cancer. AI is an advanced form of technology that uses mathematical-based algorithmic principles to face complex challenges in healthcare (8).

Multi-omics integrates genomics, transcriptomics, proteomics, and metabolomics to uncover molecular signatures driving diseases like cancer (9). Emerging technologies, including single-cell RNA sequencing and microfluidics-based detection of circulation tumor DNA (ctDNA), exosomes, and circulation tumor cells (CTCs), provide high-resolution profiling of tumors and their microenvironments, enabling more accurate identification of tissue-of-origin in cancers of CUP (10). Most modern cancer therapies particularly targeted agents are selected according to the known site of origin. However, a subset of patients (historically 3-5%) present with metastatic disease which standard diagnostic workup fails to identify the primary tumor, resulting in CUP. These tumors typically behave aggressively and are associated with poor outcomes, with median survival generally under one year. Empiric chemotherapy has been the conventional approach but provides limited benefit for many patients. Consequently, improved diagnostic strategies are needed to accelerate and refine tissue of origin identification and enable more rational, biomarker informed treatment (1).

AI-powered MP leverages machine learning to analyze complex multi-omics data, enhancing diagnostic precision and treatment personalization in cancers of CUP (9, 11). Tools such as CUP-AI-DX and whole-genome sequencing approaches exemplify how computational models can combine omics and clinical data to infer tumor origin and inform therapeutic decisions. Recent by Zhao et al. and Rebello et al., further emphasize the clinical utility of AI-assisted molecular profiling in CUP, supporting its role in resolving diagnostic uncertainty and guiding personalized treatment strategies (10, 12). By identifying actionable biomarkers, AI-driven MP resolves diagnostic uncertainties where traditional methods fail. Advanced bioinformatics and technologies like blockchain further support secure, scalable multi-omics analysis. These innovations are critical for advancing precision oncology and tailoring therapies to individual patients (9, 11, 13).

The integration of advanced computational tools in genomic research is pivotal for advancing precision medicine (11, 13). With clinical laboratories performing billions of tests each year, contributing to the majority of medical decisions, the demand for precise and comprehensive data is critical. Leveraging artificial AI to create health indices, forecast disease progression, and combine sophisticated statistical models with digital twin technologies demonstrates the potential to transform healthcare delivery (11, 14, 15). However, challenges such as deriving actionable insights from complex omics data and establishing robust comparative metrics must be addressed to fully realize these benefits. As these innovations redefine medical practice, ethical considerations are crucial to ensure that technology enhances, rather than overshadows, the human element in patient care. The future of medicine hinges on a nuanced understanding of patient experiences and care pathways, aligning AI-driven advancements with the intricacies of individual health to promote patient-centered outcomes (9, 11, 13–16).

Considering the advancements in cancer diagnosis and treatment research, the use of AI-powered MP has shown a promising role as a decision-support aid for physicians. It helps to deliver personalized care for cancer patients. MP of tumor DNA assesses the presence of genetic alterations in genes and guides treatment based on individuals’ tumor profile. Tumor profiling ranges from examining specific genetic mutations to sequencing many genetic alterations, including amplifications, deletions, insertions, duplications, substitutions, structural variants, inversions, translocations as well as gene expressions. As previously mentioned, one of the crucial advancements in sequencing technologies is the NGS technique, which significantly impacted the understanding of genetic mutations and created a tremendous revolution in genomic research. NGS allows the ability to test multiple genes simultaneously, making this technology a handy tool in clinical practice and research.

The use of MP in assessing genetic alterations in an individual’s tumor may provide treatment guidance based on an extensive literature review and identify clinical trials relevant to specific biomarkers. Each molecular profile is linked to published evidence regarding the same biomarker.

Guided treatments via MP of solid tumors using specific biomarkers have resulted in better outcomes in patients with certain mutations, such as those with EGFR (Epidermal Growth Factor Receptor) mutant lung cancer and BRAF mutant melanoma (17). Various organizations now provide MP platforms to guide physicians in their therapeutic decisions in metastatic and early disease.

This report assesses the significance of using MP as a decision support aid for physicians. It provides them with a list of possibly actionable genetic biomarkers to help tailor treatment and improve clinical outcomes in patients with CUP. Initial clinical and histopathological evaluations suggested the possibility of recurrent prostate cancer or a new primary pancreaticobiliary malignancy. However, utilizing MP provided diagnostic insights that revealed a different primary cancer altogether. This case emphasizes the necessity of incorporating comprehensive molecular diagnostics in routine clinical practice, especially for patients with complex oncological histories. It also highlights the potential for these technologies to uncover rare and unexpected diagnoses, facilitating more effective and personalized treatment strategies.

## Case presentation

This is a 69-year-old male patient who first presented with mild to moderate difficulties in erection and sexual activity with a notable medical history including coronary artery disease, managed with two cardiac stents, stage III chronic kidney disease, and type II diabetes mellitus. History revealed an International Index of Erectile Function (IIEF-5) of 4, indicating mild to moderate difficulties in erection and sexual activity. The American Urological Association (AUA) Symptom Index was 7, indicating mild urinary difficulties. His family history was negative for prostate cancer. The patient had elevated Prostate-specific antigen (PSA) levels and underwent a diagnostic biopsy of the prostate, which revealed low-grade, low-volume adenocarcinoma in a single core, with a Gleason score of 6 (3 + 3) involving 10% of the sampled tissue, without perineural invasion or extracapsular extension and remained under active surveillance. The patient had continued elevation in PSA levels and underwent a second confirmatory core biopsy of the prostate, which revealed similar results and active surveillance was continued per patient preference. The patient had increasing symptoms and underwent a High-intensity focused ultrasound (HIFU) of the prostate 12 months from the onset of symptoms, which provided symptomatic relief.

After four months, he had elevated PSA. Follow-up imaging remained clear, and the patient’s PSA levels stabilized between 2–3 ng/mL, indicating a state of Complete Remission (CR) for 24 months. Then, he had recurrent symptoms, including hematuria, with PSA levels increased to 4.2 ng/mL, prompting further investigation for recurrent prostate cancer. Magnetic Resonance Imaging (MRI) and Prostate-specific Membrane Antigen Positron Emission Tomography (PSMA-PET) scans revealed local recurrence and multiple osseous metastases. The patient then underwent bronchoscopy cytology which revealed adenocarcinoma of unknown origin (did not definitively identify the renal lesion as a primary tumor). Treatment with darolutamide and relugolix was initiated and yielded an excellent PSA response. However, after four months of therapy initiation, imaging subsequently identified new metastatic lesions in the lungs and liver. However, the abdominal ultrasound and a biopsy revealed poorly differentiated carcinoma with possible pancreaticobiliary origin. Thus, the patient was given five cycles of carboplatin and cabazitaxel per The National Comprehensive Cancer Network (NCCN) guidelines. Immunohistochemistry (IHC) stains showed that tumor cells were strongly positive for CK7 and negative for TTF1, P40, NKX3.1, CDX2, GATA3, STAB2, and CK20. These features did not support cancers originating from the lungs, prostate, urinary tract, or gastrointestinal tract. Treatment with carboplatin and cabazitaxel was stopped, and the patient had normal CA 19–9 levels. After three months, the patient underwent genetic testing by Caris Life Sciences, which revealed deletions of genetic ATM, CDKN2A, and MTAP, alongside alterations in the BARD1 gene, possibly indicating the presence of other primary tumors. The tumor was microsatellite stable with a Tumor Mutational Burden (TMB) of 4 mutations per megabase and negative for androgen receptor expression, ALK fusion, NTRK1/2/3, and RET fusion.

Tumor markers were unremarkable, including Cancer-Embryonic adenocarcinoma (CEA), Alpha-Fetoprotein (AFP), PSA, and CA 19-9. NGS for MP and tissue typing was sent out, and the patient was started on gemcitabine, cisplatin, and durvalumab for a suspected pancreaticobiliary tumor. The patient’s predominant symptom was a persistent cough. Subsequent Computed Tomography (CT) imaging revealed an enlarging lytic lesion in the left superior acetabulum. At the same time, the widespread osseous, pulmonary, hepatic, adrenal, and nodal disease remained stable relative to prior PET/CT findings.

The patient’s tumor tissue was analyzed using Caris Life Sciences’ AI-powered-MI platform, a multi-omics profiling tool. This platform integrates whole exome sequencing (WES) of more than 22,000 DNA genes, whole transcriptome sequencing (WTS) of more than 22,000 RNA genes, and immunohistochemistry IHC to provide a comprehensive molecular profile. Germline variants are bioinformatically filtered using established database and variant annotation pipelines to enrich for somatic tumor-specific alterations prior to molecular interoperation.

The Caris platform uses AI-driven algorithms (GPSai), including machine learning techniques such, to predict tumor tissue of origin by comparing the patient’s molecular signature against a reference database of thousands of tumors, guiding diagnosis and therapeutic decisions. GPSai is a supervised machine-learning-based tissue-of- origin classifier trained on a large reference database of molecularly an noted tumors integrating genomic and transcriptomic features. Published analytical and clinical validation studies have demonstrated high diagnostic accuracy across multiple tumor types.

In this case, GPSai predicted an 82% chance of RCC, 9% chance of urothelial carcinoma, and 3% chance of Hepatocellular Carcinoma (HCC). The reported percentage represents a probabilistic classification score generated by the supervised machine-learning model, reflecting the degree of molecular similarity between the patient’s tumor profile and validated reference tumor datasets. This probability is derived from comparison across multiple cancer types included in the training cohort and has undergone analytical and clinical validation in peer-reviewed studies evaluation tissue-of- origin prediction performance. The GPSai model is not FDA-approved as a diagnostic tool; however, its analytical and clinical validity has been demonstrated I peer-reviewed studies, supporting its reliability in identifying tumor tissues of origin.

**Figure 1:**
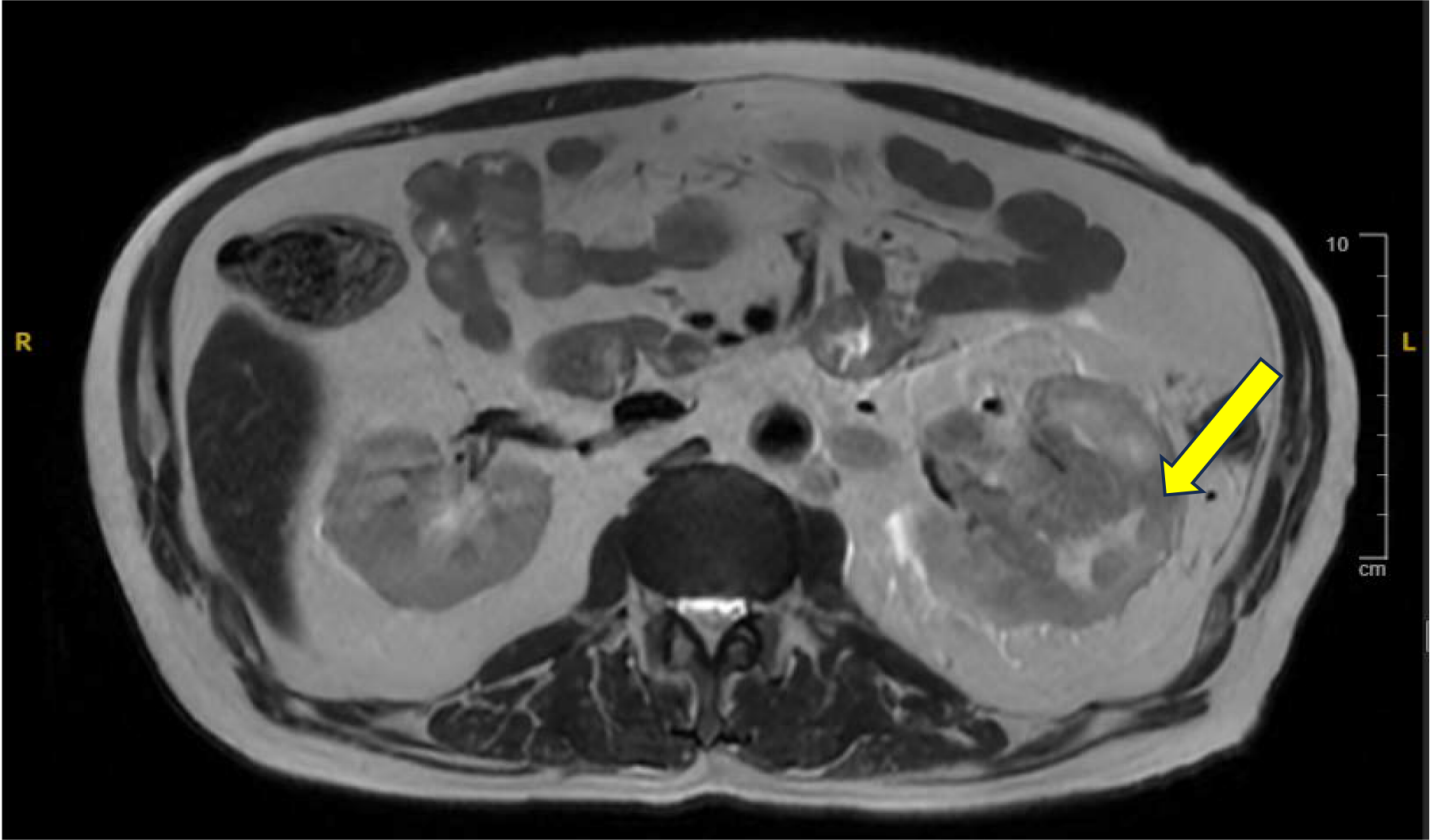
Axial contrast-enhanced MRI showing a heterogeneous enhancing mass in the left kidney (arrow), subsequently confirmed as renal cell carcinoma with sarcomatoid differentiation on biopsy.

**Figure 2:**
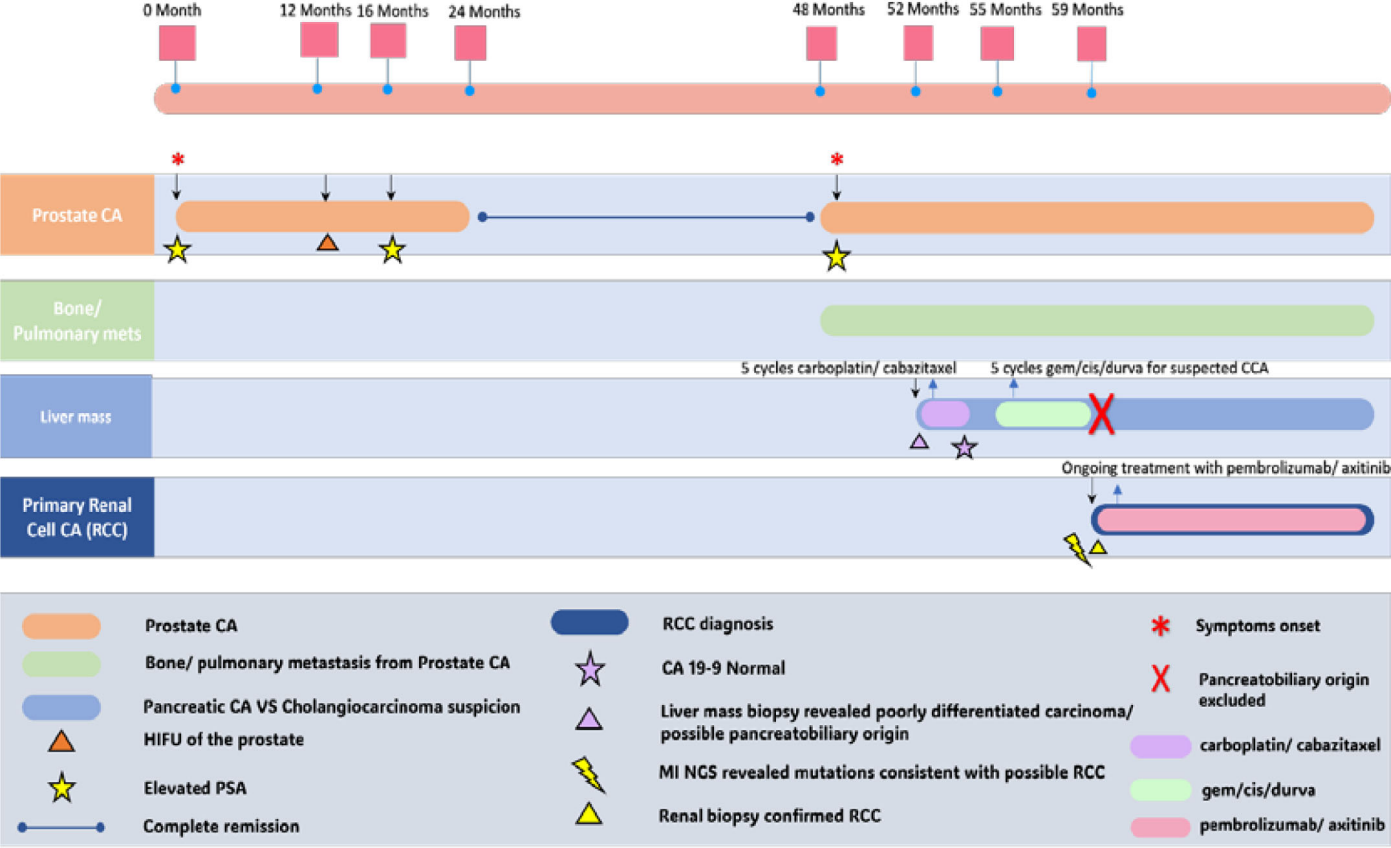
Chronological timeline of the patient’s clinical course, including initial prostate cancer diagnosis, metastatic progression, prior treatments (HIFU, systemic chemotherapy), suspicion of pancreaticobiliary malignancy, AI-based molecular profiling (GPSai: 82% likelihood of RCC), renal biopsy confirmation, and subsequent targeted therapy with pembrolizumab and axitinib.

The patient had gross hematuria, and an abdominal MRI showed a suspected left renal lesion (**Figure 1**). Due to these results, the gemcitabine, cisplatin, and durvalumab regimen were stopped, and a deeper investigation for a primary renal mass commenced. A biopsy of a left renal mass confirmed the diagnosis of RCC with focal sarcomatoid differentiation and the International Society of Urological Pathology (ISUP) grade of 4/4. The patient was started on a combination of pembrolizumab and Axitinib and has completed three cycles of this regimen as of the time of this report. Treatment evaluation scans post four cycles are pending. A timeline with detailed information about the patient’s diagnostic and treatment history is shown in **Figure 2**.

These findings underline the importance of utilizing MI and NGS in the early diagnosis and treatment of cancers with CUP. Giving patients an earlier optimal therapy may provide better clinical benefits.

## Discussion

Using the patient’s clinical and molecular data is crucial for providing a personalized therapeutic approach in the patient’s best interest. Variable molecular information is available due to NGS techniques, which increases the probability of identifying the truly distinguishable molecular features among different cancers. AI systems can learn the associations between data by just having the examples and catch non-linear relationships among the data (18).

AI-MP platforms, like Caris Life Sciences’ MI, use machine learning to integrate multi-omics data, achieving high diagnostic accuracy (94% for 21 cancer types) in cancers of CUP. In this case, MI’s MI GPSai™ identified RCC with 82% likelihood. Non-AI tools, rely on targeted NGS or IHC, with lower accuracy (50–60%) due to limited scope. While non-AI tools are cost-effective, AI-driven MP excels in resolving CUP’s complexity, requiring robust data privacy measures (19).

The MI GPSai algorithm was previously trained and validated on more than 77,000 genomic and transcriptomic profiles and demonstrated over 94% accuracy in predicating type across 21 cancer categories in indented validation cohorts (2).

The patient in our case was diagnosed with RCC via MP after receiving multiple lines of treatment for other suspected primary tumors. The utilization of MP in diagnosing and treating CUP may be influential in the early diagnosis and management of cancer.

A recent study reported a case of a 43-year-old man with no relevant medical history who presented with progressive cough and dyspnea despite receiving treatment for pneumonia. He was found to have enlarged subcarinal, hilar, retroperitoneal, and mesenteric Lymph Nodes (LN), suspicious for metastasis, and a mass in the right kidney. Pathology reports of lymph nodes were most consistent with metastatic epithelioid angiomyolipoma (mEAML). The patient was urgently treated with a Mammalian target of rapamycin (mTOR) inhibitor after three weeks due to rapid deterioration of his suspected mEAML. Subsequently, he underwent an LN biopsy to confirm the diagnosis with comprehensive genomics, including Whole Exome and Transcriptome Sequencing (WES/WTS) alongside IHC. Unfortunately, the patient developed hypoxic respiratory failure, and only after his death did WES/WTS reveal pathogenic variants in BAP1 and VHL, consistent with clear cell RCC (ccRCC). An earlier diagnosis would have led to the initiation of optimal systemic therapy for ccRCC with potentially lower tumor burden and better clinical stability. This study concluded that following comprehensive, molecular-based genomic sequencing in rapidly progressing malignancies or CUP may be crucial to provide earlier optimal therapeutic options and lead to the best possible clinical benefit (20).

Another study was carried out by Fenstermaker et al., where they developed a Deep Learning (DL)-based algorithm for RCC diagnosis, subtyping, and grading. This DL-based algorithm reached a high level of accuracy with only a 100 square micrometers (µm2) patch, which makes it a possibly helpful tool in renal mass biopsy analysis. A computational method tested on small tissue biopsies may lower the risk of insufficient tissue sampling and decrease the need to repeat biopsies. However, this study identified the 3 main subtypes of RCC without focusing on benign renal tumors or oncocytomas, which represent a significant proportion of small renal masses (21).

There are several changes for AI-powered MP including drug prioritization in cancer research faces biological, technical, and ethical challenges, including limited expertise in multi-omics, inconsistent sample quality, and computational limitations. AI-driven genomics struggles with model interpretability, data heterogeneity, and biases, necessitating explainable AI and robust data-sharing methods like federated learning (11, 13). Ethical issues, such as data privacy, informed consent, and algorithmic bias, require comprehensive regulatory frameworks. Biologically, incomplete understanding of tumor heterogeneity and drug resistance complicates precision oncology (11). Technical hurdles, like DNA degradation in FFPE samples and missing multi-omics data, demand advanced imputation methods and standardized protocols (11).

The development of sophisticated bioinformatics platforms is critical for advancing multi-omics research (9, 16). These platforms must facilitate the smooth integration and analysis of varied omics datasets, such as genomics, transcriptomics, proteomics, and metabolomics (9, 16).The future of multi-omics research relies on advanced bioinformatics tools to integrate and analyze diverse omics datasets, supported by enhanced computational power and cloud computing. Blockchain technology offers a secure, transparent solution for managing multi-omics data, fostering trust and collaboration (9, 11, 16). Interdisciplinary partnerships between data scientists, clinicians, and researchers are crucial for translating complex multi-omics insights into clinically relevant applications. Standardized protocols and advanced statistical methods are needed to address challenges in data collection, analysis, and reproducibility. Multi-omics approaches will drive personalized medicine by tailoring treatments using integrated genetic, transcriptomic, proteomic, and metabolomic data (9, 11, 13–16). The whole genome and exome sequencing will uncover novel biomarkers and drug targets for complex diseases. Emerging technologies, including AI-driven virtual patient models and improved data visualization, will enhance precision medicine over the next decade.

However, several limitations must be acknowledged. First, the diagnostic accuracy of AI-powered MP may vary depending on the platform used as different platforms employ distinct algorithms, reference datasets, and sequencing coverage, potentially affecting tissue-of-origin predictions. For example, Zhao et al. developed as RNA-seq-based tool (CUP-AL-Dx) trained across 32 cancer types, achieved 98.5% accuracy in cross-validation and 96.7% in internal test sets, but performance dropped to 86.9% and 72.5% when applied to independent clinical-grade datasets from the US and Australia, respectively. When applied to a balanced clinical series, accuracy decreased to 75.5% with misclassification most pronounced among pancreatobiliary tumors, highlighting challenges in tumors with overlapping histology. These observations underscore the impact of tumor heterogeneity biopsy site contamination, and platform differences on predictive performance (10).

Second, the patient’s prior history of prostate cancer and multiple treatments, including HIFU and chemotherapy, may have influenced tumor molecular profiles, potentially confounding AI predictions. Moreover, AI models are trained on populations that may not fully represent patients with complex oncologic histories, limiting generalizability. These observations underscore the importance of integrating AI-MP results with clinical, pathological, and imaging data rather than relying solely on computational predictions.

Overall, AI-driven multi-omics profiling represents a powerful approach to resolving CUP complexity, but its application must consider biological, technical, and platform-related limitations. Continued development, validation, and integration of multi-omics data, including SNVs, transcriptomics, and histopathology, will be essential to enhance diagnostic precision and guide optimal therapy.

## Conclusion

Our case showed that MP played a crucial role in optimizing patient care. MP can provide critical information about tumors and guidance for physicians to implement optimal medical treatment for patients with CUP. However, continued research is required to validate the importance of utilizing MP in the diagnosis and treatment of patients with CUP.

## Data Availability

The raw data supporting the conclusions of this article will be made available by the authors, without undue reservation.

## References

[1] Chhikara BS Parang K . Global Cancer Statistics 2022: the trends projection analysis. Chem Biol Lett. (2023) 10:451–1.

[2] Kato S Kim SY Lim HJ Boichard A Nikanjam M Sicklick JK . Multi-omic analysis in carcinoma of unknown primary (CUP): therapeutic impact of knowing the unknown. Mol Oncol. (2024) 18:956–68. doi: 10.1002/1878-0261.13293, PMID: 35866362 PMC10994241

[3] Rassy E Pavlidis N . The currently declining incidence of cancer of unknown primary. Cancer Epidemiol. (2019) 61:139–41. doi: 10.3389/fonc.2019.01546, PMID: 31254795

[4] Kolling S Venturini N Jørgensen JT Steiniche T Nielsen AL Bogsted M . Metastatic cancer of unknown primary” or “primary metastatic cancer”? Front Oncol. (2020) 9:1546. doi: 10.3389/fonc.2019.0154, PMID: 32010631 PMC6978906

[5] Malone ER Oliva M Sabatini PJB Stockley TL Siu LL . Molecular profiling for precision cancer therapies. Genome Med. (2020) 12:1–19. doi: 10.1186/s13073-019-0703-1, PMID: 31937368 PMC6961404

[6] Abraham J . Leveraging artificial intelligence to find previously undiscovered patterns. In: Tumor molecular data to aid in diagnosis and therapy selection. Tempe, Arizona, USA: Arizona State University (2020).

[7] Dawood S Natarajan V Danchaivijitr P . Comprehensive molecular profiling identifies actionable biomarkers for patients from Thailand and the United Arab Emirates with advanced Malignancies. Front Oncol. (2024) 14:1374087. doi: 10.3389/fonc.2024.1374087, PMID: 38800398 PMC11116666

[8] Sufyan M Shokat Z Ashfaq UA . Artificial intelligence in cancer diagnosis and therapy: Current status and future perspective. Comput Biol Med. (2023) p:107356. doi: 10.1016/j.compbiomed.2023.107356, PMID: 37688994

[9] Chen C Zou Y Chen J Gong Z Tang H Wen Z . Applications of multi-omics analysis in human diseases. MedComm. (2023) 4:e315. doi: 10.1002/mco2.315, PMID: 37533767 PMC10390758

[10] Zhao Y Pan Z Namburi S Kanneganti A Low S Ittmann M . CUP-AI-Dx: A tool for inferring cancer tissue of origin and molecular subtype using RNA gene-expression data and artificial intelligence. EBioMedicine. (2020) 61:103030. doi: 10.1016/j.ebiom.2020.103030, PMID: 33039710 PMC7553237

[11] Srivastava R . Advancing precision oncology with AI-powered genomic analysis. Front Pharmacol. (2025) 16. doi: 10.3389/fphar.2025.1591696, PMID: 40371349 PMC12075946

[12] Rebello RJ Kusnadi EP Cameron DP Lin D Walker SM Gurney H . Whole genome sequencing improves tissue-of-origin diagnosis and treatment options for cancer of unknown primary. Nat Commun. (2025) 16:4422. doi: 10.1038/s41467-025-59661-x, PMID: 40393956 PMC12092688

[13] Srivastava R . Applications of artificial intelligence in medicine. Exploratory Res Hypothesis Med. (2024) 9:138–46. doi: 10.14218/ERHM.2023.00048, PMID: 39130623

[14] Ahmed Z . Practicing precision medicine with intelligently integrative clinical and multi-omics data analysis. Hum Genomics. (2020) 14:35. doi: 10.1186/s40246-020-00287-z, PMID: 33008459 PMC7530549

[15] Bakshi B Pugliese P Al-Saffar M Griffiths M Jha A Perera R . Using an artificial intelligence platform to enhance cancer detection rates in primary care. Am Soc Clin Oncol. (2024) 42(16_suppl):1560. doi: 10.1200/JCO.2024.42.16_suppl.1560, PMID: 41735675

[16] Correa-Aguila R Alonso-Pupo N Hernández-Rodríguez EW . Multi-omics data integration approaches for precision oncology. Mol Omics. (2022) 18:469–79. doi: 10.1039/D1MO00411E, PMID: 35470819

[17] Carter P Schwaederle M Patel SP Janku F Naing A Miller V . Does molecular profiling of tumors using the Caris molecular intelligence platform improve outcomes for cancer patients? Oncotarget. (2018) 9:9456. doi: 10.18632/oncotarget.24258, PMID: 29507702 PMC5823623

[18] Giulietti M Piva F Scirè ME Principato G Santoni M Mocchegiani F . The role of artificial intelligence in the diagnosis and prognosis of renal cell tumors. Diagnostics. (2021) 11:206. doi: 10.3390/diagnostics11020206, PMID: 33573278 PMC7912267

[19] Yates J Van Allen EM . New horizons at the interface of artificial intelligence and translational cancer research. Cancer Cell. (2025) 43:708–27. doi: 10.1016/j.ccell.2025.03.018, PMID: 40233719 PMC12007700

[20] McLoughlin DE Choo-Wosoba H Gills JJ Peer CJ Figueroa NM Cordes LM . A rare presentation of aggressive renal cell carcinoma and the utility of early molecular testing in rapidly progressing Malignancies: A case report. Oncologist. (2023) 28:1094–9. doi: 10.1093/oncolo/oyad280, PMID: 37844295 PMC10712707

[21] Fenstermaker M Tomlins SA Singh K Hafez KS Miller DC Montgomery JS . Development and validation of a deep-learning model to assist with renal cell carcinoma histopathologic interpretation. Urology. (2020) 144:152–7. doi: 10.1016/j.urology.2020.05.094, PMID: 32711010

